# New bone formation of biphasic calcium phosphate bone substitute material: a systematic review and network meta-analysis of randomized controlled trials (RCTs)

**DOI:** 10.1186/s40729-025-00636-4

**Published:** 2025-07-15

**Authors:** Chutikarn Somngam, Sutiwat Samartkit, Sukrit Kanchanasurakit, Frank Peter Strietzel, Pathawee Khongkhunthian

**Affiliations:** 1 Center of Excellence for Dental Implantology, Faculty of Dentistry, Chiang Mai University, Chiang Mai, 50200 Thailand; 2 Center of Health Outcomes Research and Therapeutic Safety (Cohorts), School of Pharmaceutical Sciences, University of Phayao, Phayao, Thailand; 3 Unit of Excellence on clinical Outcomes Research and Integration (UNICORN), School of Pharmaceutical Sciences, University of Phayao, Phayao, Thailand; 4 Division of Clinical Pharmacy, Department of Pharmaceutical Care, School of Pharmaceutical Sciences, University of Phayao, Phayao, Thailand; 5 Division of Pharmaceutical care, Department of Pharmacy, Phrae Hospital, Phrae, Thailand; 6 Department of Oral Medicine, Dental Radiology and Oral Surgery, Charite Centre 3 for Dental, Oral and Maxillary Medicine, Charite – Medical University of Berlin, Berlin, Germany

**Keywords:** Dental implants, Sinus floor augmentation, Hydroxyapatite-beta tricalcium phosphate, Biphasic calcium phosphate, BCP, Histomorphometry, Network meta-analysis

## Abstract

**Objective:**

• To systematically determine the effectiveness of biphasic calcium phosphate (BCP) as bone substitute materials (BSM) compared to other BSMs for new bone formation in dental implant treatment through a network meta-analysis (NMA).

**Materials and method:**

• Following PRISMA-NMA guidelines, randomized controlled trials (RCTs) on lateral sinus augmentation and dental implants comparing BCP with other BSMs for histomorphometric new bone formation were included. Studies were retrieved from MEDLINE, Cochrane, Scopus, and Embase (up to November 2024), with quality assessed via the Cochrane risk of bias 2 (RoB2.0) tool. Analysis included direct and network meta-analyses using a random-effects model, with SUCRA scores determining treatment rankings. The PROSPERO registration number was CRD42024607526.

**Results:**

• Of 268 studies, 11 met criteria, covering 283 patients and 362 sinus augmentations using autografts (AB), allografts (AL), beta tricalcium phosphate (TCP), BCP, and xenografts (Xeno). NMA showed AB resulted in 12.33% more new bone formation than BCP (95% CI: 10.74, 13.93), with AL showing 5.14% more (95% CI: 3.33, 6.95). Xeno showed 4.14% less bone formation than BCP (95% CI: -6.38, -1.90). AB ranked highest for new bone formation, followed by AL, BCP, TCP, and Xeno. Residual graft material was highest in Xeno (6.21%; 95% CI: 2.81, 9.61).

**Conclusion:**

• BCP demonstrated sufficient new bone formation, outperforming xenografts in both bone formation and residual graft material. While autografts and allografts exhibited superior bone regeneration, BCP remains an effective option for bone augmentation treatments.

**Supplementary Information:**

The online version contains supplementary material available at 10.1186/s40729-025-00636-4.

## Background

After tooth extraction, dimensional changes in bone and soft tissue inevitably take place during the healing process [1–3]. Consequently, inadequate alveolar ridge height and width can pose challenges when dental implant treatment is required. One of the procedures used to increase bone volume is bone augmentation using bone substitute materials (BSM) [4, 5]. Autogenous bone is currently considered as a gold standard graft material since it can provide essential properties for bone formation: osteoinductivity, osteogenecity, and osteoconductivity [6–9]. However, harvesting bone from the donor site requires an additional surgery, which can cause patient morbidity [8–12]. Moreover, the amount of bone obtained is limited, and the graft’s resorption rate is variable [7, 10, 11]. These drawbacks of autografts have led to the development of new materials, including alloplasts, allografts, and xenografts [7, 8, 10, 12].

Alloplasts, synthetic BSM, are developed to overcome the drawbacks of natural bone grafts. Although designed to mimic the structural and biological properties of human bone, alloplasts typically exhibit mainly osteoconductive properties [7, 13]. Current synthetic materials can be categorized into bioceramics and polymers [13–15]. Biphasic calcium phosphate (BCP), one of pure synthetic bioceramic materials, is the combination of beta-tricalcium phosphate (TCP) and hydroxyapatite (HA) [9, 13, 14, 16–18]. Combining TCP with HA allows it to achieve optimal mechanical strength and resorption rates [8, 9, 12–14]. With this combination, BCP is enhanced in mechanical properties by the strength of HA and provides greater and faster resorption rate than HA alone due to the role of TCP [12, 13, 16, 18]. Moreover, the osteoconductive activity and biodegradability of BCP can be altered by adjusting the proportion of HA/TCP [13, 19]. For instance, BCP with a 60:40 HA/TCP ratio has more residual graft material at follow-up than the 20:80 ratio, indicating slower resorption and greater structural stability [20]. In contrast, the 20:80 HA/TCP ratio demonstrates superior bone regenerative properties compared to the 60:40 ratio and HA alone, as evidenced by a higher amount of bone formation [20, 21]. In addition to its osteoconductive capabilities, BCP also exhibits osteoinductivity which is essential for bone healing [13, 22]. Since calcium ions (Ca^2+^) play an important role in stimulating and maturation of pre-osteoblast into osteoblast cells, which indicate the osteoinduction activity of grafts. The higher Ca^2+^ concentration released by BCP implies greater osteoinductivity compared to HA alone. In particular, the 20:80 HA/TCP ratio has been shown to release more Ca²⁺ than HA alone, contributing to increased new bone formation [21, 22].

Allografts BSM deriving from donors of the same species, either living humans or cadavers, are used in various procedures, including periodontal and alveolar bone reconstruction [7, 9, 10, 13]. However, the risk of disease transmission, potential immune response, increasing supply shortages, and increased regulatory restrictions (particularly in Europe) limit their clinical use and have led to a gradual shift toward other bone graft substitutes [7 – [10, 13]. Allografts can be obtained in three forms. Fresh-frozen bone allografts (FFB) provide highest osteoinductive activity but are rarely used due to risks of immune response, limited shelf life, and disease transmission [7, 13]. Freeze-dried bone allografts (FDBA) are mainly osteoconductive and have inferior osteoinductive potential than FFB. Demineralized freeze-dried bone allografts (DFDBA) has osteoinductive properties, but it lacks structural support due to the removal of the mineral component [7, 9, 13].

Xenografts derive from non-human species donors, which deproteinized bovine bone is the most commonly used xenograft. Deproteinized bovine bone has been treated by heat and chemical processes to remove organic components causing immunoreaction and disease transmission, result in being only osteoconductive and presenting mainly a porous hydroxyapatite (HA) structure resembling human bone [7, 9, 13].

However, like many other newly developed materials, there still are a small number of documented reports of clinical uses of BCP as bone substitutes in humans [10, 23–25]. This scarcity of evidence, along with the growing number of available bone substitute materials (BSMs), presents a challenge for direct comparisons. Traditional pairwise meta-analyses are limited to direct comparisons and the synthesis of results between two interventions across different trials. However, when head-to-head comparisons are scarce, pairwise meta-analyses are unable to incorporate the broader body of available evidence across multiple interventions. In contrast, network meta-analysis (NMA) enables the comparison of three or more interventions by integrating both direct and indirect evidence, allowing for the simultaneous evaluation of multiple treatments within a single analytical framework [26–28]. Given the wide variety of BSMs developed in recent years, NMA offers a suitable approach to compare the effectiveness of BCP not only against individual comparators, but across all BSMs studied to date. Therefore, the purpose of this meta-analysis was to systematically determine the histomorphometric effectiveness of BCP as BSM in comparison to other bone grafts in dental implant treatment in term of new bone formation, through a network meta-analysis (NMA).

## Materials and methods

### Protocol and registration

A research protocol was developed according to the Preferred Reporting Items for Systematic Reviews and Meta-Analyses (PRISMA) extension statement for NMA [29]. This meta-analysis was registered under the trial number CRD42024607526 in the International Prospective Register of Systematic Reviews (PROSPERO: www.crd.york.ac.uk/PROSPERO).

### Eligibility criteria

This study included randomized controlled trials (RCTs) in which patients were treated with lateral sinus augmentation and dental implant procedures. Given the objective of this study to evaluate the histomorphometric outcomes of BCP, the surgical technique was limited to lateral sinus augmentation, as it reliably allows for the retrieval of histological samples. This approach was selected as it provides predictable clinical results [24, 25, 30], allowing for consistent histomorphometric comparison of BSMs. Moreover, surgical variables such as membrane coverage and Schneiderian membrane perforation have been reported not to significantly affect the success rate of the procedure [23, 31–34], supporting the validity of this focused approach. Additionally, Restricting the analysis to a single intervention also improves the transitivity assumption of the network meta-analysis.

Only studies using BCP compared with other BSMs and reporting histomorphometric outcomes as percentages of new bone formation were included. The length of the follow-up period for each trial was not limited. Publications other than RCTs (e.g., reviews, meta-analysis, clinical study, retrospective study) were excluded from the analysis. Additional exclusion criteria included studies that did not report new bone formation outcomes as percentages or those whose data were insufficient for inclusion in the NMA.

### Literature search strategy

The electronic search was done in MEDLINE via PubMed, Cochrane, Scopus, and Embase databases using combinations of the following search terms: Dental implants/ Dental implantation, Sinus floor augmentation/ Alveolar ridge augmentation/ Alveolar bone grafting, Hydroxyapatite-beta tricalcium phosphate (HA/ β-TCP)/ Biphasic calcium phosphate (BCP). The literature search was restricted to English-language studies and conducted on human subjects. The online search started from inception until 25 November, 2024. Detailed search strategies for each database are provided in Supplementary Table S1.

### Study selection and data extraction

Excluding duplicated studies and selecting studies to retrieve potentially eligible studies by screening the titles and abstracts were done by two independent reviewers (CS and SS) using the Rayyan web application. Any conflicts between two reviewers (CS and SS) were discussed, and if consensus could not be reached, a third reviewer (PK) acted as an adjudicator and made the final decision.

Full texts of all relevant publications were re-evaluated according to the eligible criteria. To maintain our main objective in evaluation the true effectiveness of BCP compared to other BSM in terms of new bone formation, studies that combined different types of BSM or combined BSM with additive substance, defined as combined materials in this study, were excluded.

The data were then extracted from the included studies using a standardized data extraction form to collect key information from each study, including the first author and year of publication, study design, type of BSM, number of participants and specimens per group, participant characteristics, healing period, and treatment outcomes.

### Quality assessment

The quality of included studied was individually assessed by two independent reviewers (CS and SS) using instructions from the Cochrane risk of bias (RoB2.0) tool for RCTs [35]. Any disagreement would be determined by third reviewer (PK). There are 5 domains which were assessed in this tool: Bias arising from the randomization process, Bias due to deviations from intended interventions, Bias due to missing outcome data, Bias in measurement of the outcome, Bias in selection of the reported result. The overall risk-of-bias determination would be based on the worst outcome of the domains.

### Outcomes and definitions

The histomorphometrically estimated percentages of new bone formation defined in the included studies for each material were the primary outcomes. Additional outcomes reported in the publications (e.g., histomorphometric percentages of residual graft material, post-operative bone height, later bone loss, implant success/ survival rate etc.) were also retrieved. Outcome measurements were recorded as mean values and standard deviations (SD). When averages and variations were not reported as means and SD (e.g., medians, SEs, confidence intervals, interquartile ranges and ranges), statistical transformations were applied to convert the outcomes. In case where the results of each group were reported for multiple regions, a statistical method was used to combine the subgroup results into a single value [36].

### Statistical analysis for NMA

Pairwise meta-analyses of all directly compared interventions were firstly investigated using the DerSimonian and Laird random effects model to evaluate the statistical heterogeneity of studies within each comparison [28, 37]. I-squared statistic and Chi-squared statistic were used to evaluate the heterogeneity in each pairwise comparison, with heterogeneity considered significant when the *p*-value was less than 0.1.

A random-effects NMA then was conducted using Stata Statistical Software V14.1 (StataCorp LP, College Station, TX, United States) via the network command. A network graph would be represented in nodes and lines to demonstrate the connection between interventions. Each intervention would be represented by a node on the network map. The size of the nodes corresponds to the number of studies or participants conducted on each material. Lines represent available direct comparisons between interventions in randomized controlled trials (RCTs). Thicker lines indicate a greater number of studies conducted on those comparisons [28, 38].

The assumption of transitivity was assessed by verifying that the included studies had comparable eligibility criteria, patient characteristics, and surgical protocols across treatment comparisons. The global inconsistency tests were used to assess statistical differences between direct and indirect comparisons, indicating inconsistency if the *p*-value was less than 0.05. Local inconsistency was further examined using the node-splitting method. Sensitivity analyses were planned to explore the robustness of the results in relation to potential effect modifiers, such as smoking status, different HA/TCP compositions of BCP, and variations in sinus morphology (e.g., width and residual bone height). Small-study effects and publication bias were also evaluated using a comparison-adjusted funnel plot [28, 38]. The surface under the cumulative ranking curve (SUCRA) was used to rank the probability hierarchy of interventions, with larger values indicating better ranks. A league table presenting relative effectiveness and a forest plot were also prepared. Two-sided statistical testing was performed. A statistical significance was indicated if *p*-values were less than 0.05.

## Results

**Fig. 1:**
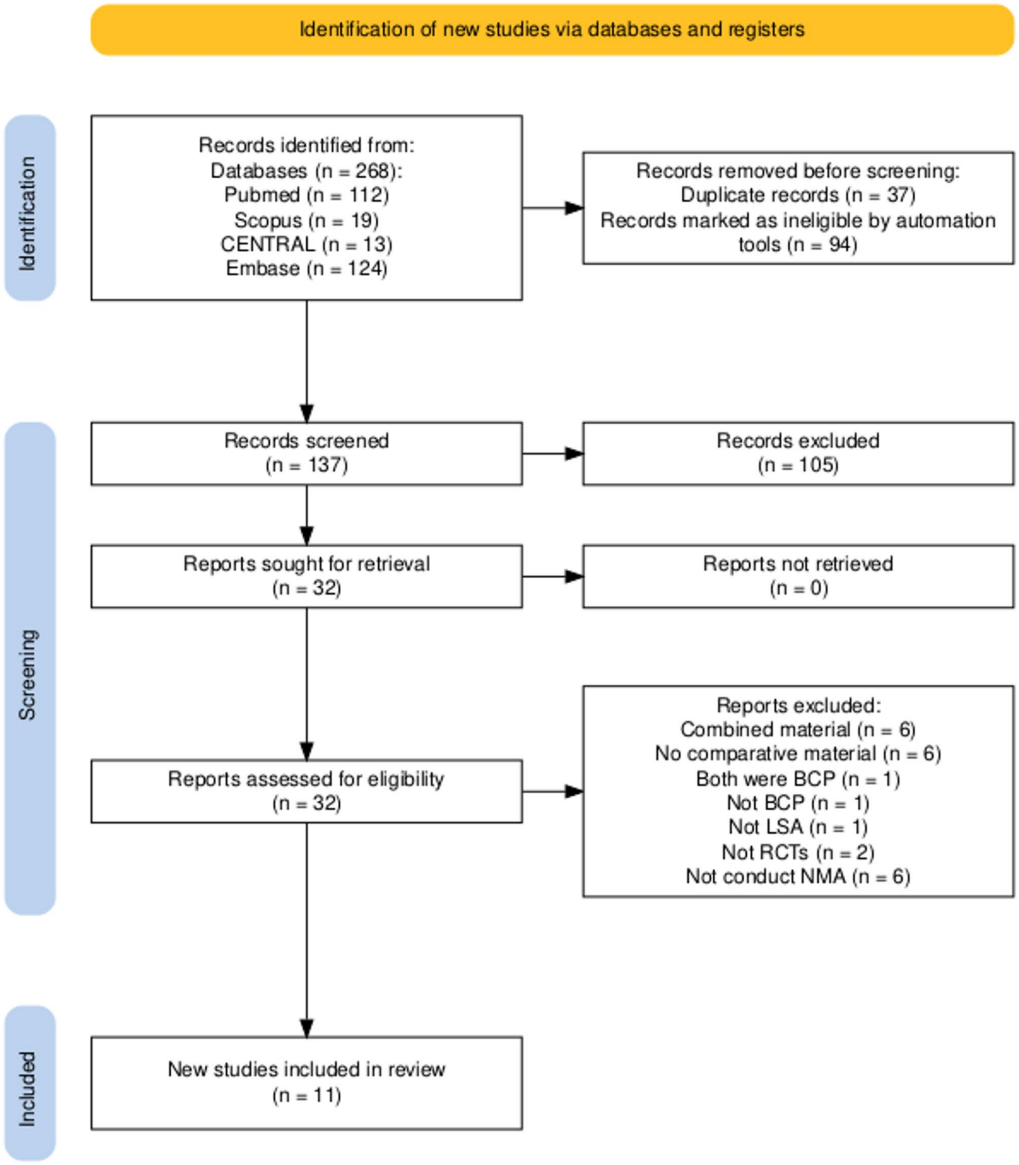
Flow diagram depicts the searching and studies selection process [58]

Out of 268 studies identified from database searching, 131 studies were removed before screening due to duplication (*N* = 37) and ineligibility (*N* = 94) via the Rayyan web application. The titles and abstracts were further screened, resulting in the exclusion of 105 publications. A total of 32 full-text articles were further analyzed, and data were extracted. However, 21 studies were excluded for the following reasons, with some studies meeting multiple exclusion criteria: use of combined materials [39–44], lack of comparative material [43, 45–49], both comparative and control groups using BCP [20], insufficient data to conduct NMA [21, 44, 50–53], use of materials other than BCP [54], no lateral sinus augmentation [55], no RCTs [56, 57]. A complete list of excluded studies with corresponding reasons is provided in Supplementary Table 2. Consequently, 11 studies met the inclusion criteria for analysis and underwent inconsistency factor testing for conducting NMA. Figure 1 depicts the PRISMA flow diagram for searching and study selection process.

### Characteristics and quality of the included studies

The materials used were autografts (AB), allografts (AL), beta tricalcium phosphate (TCP), BCP, and xenografts (Xeno). All allografts in the analysis were freeze-dried bone allograft (FDBA), and all xenografts were deproteinized bovine bone (DBB). A total of 283 patients with 362 sinus floor augmentations / specimens, within a healing period of five to ten months, were included and analyzed. Table 1 summarizes the characteristics and demographic data of the included studies, while Table 2 presents their treatment outcomes.

**Fig. 2:**
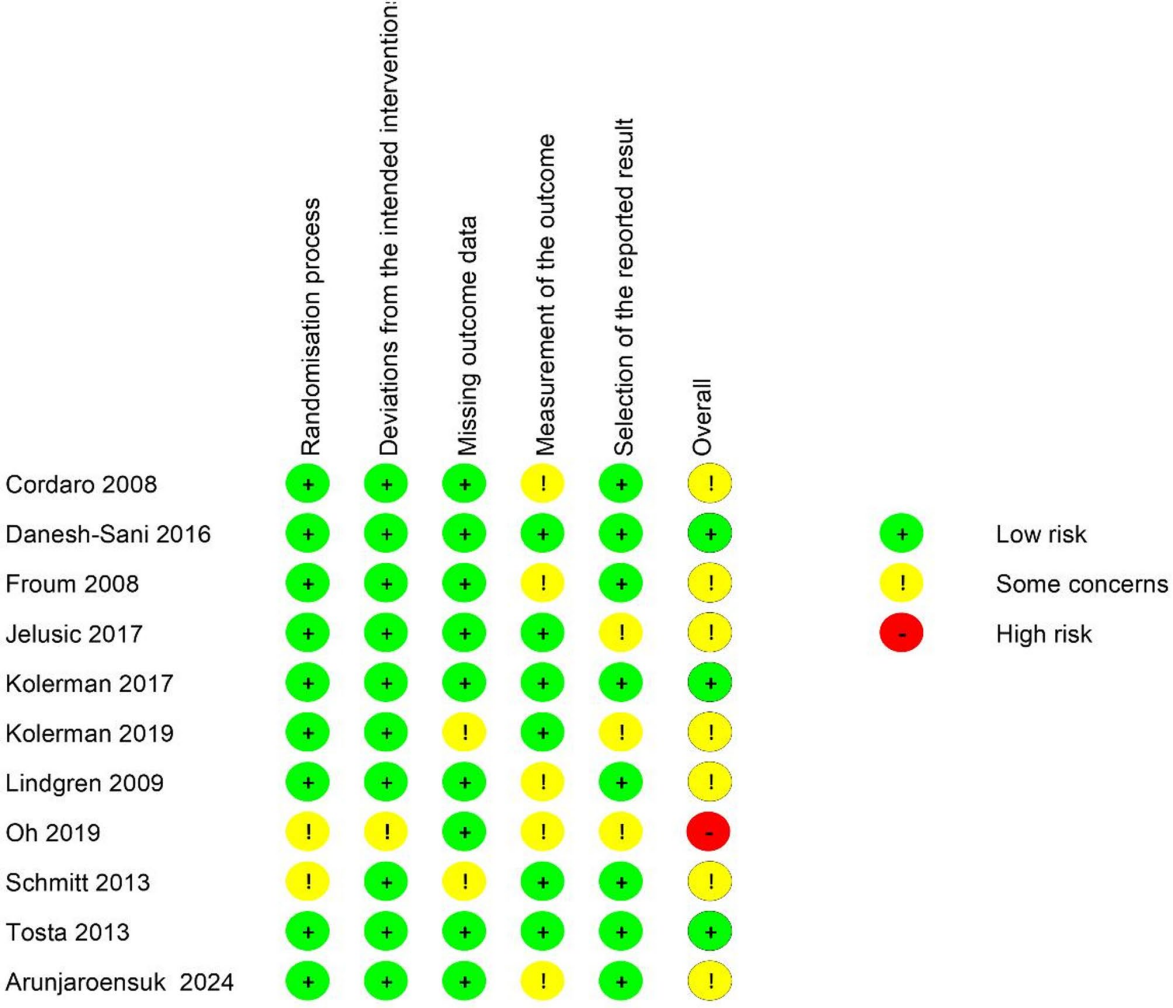
Risk of bias of individual study

The risk of bias assessment for each individual study is illustrated in Fig. 2. Of the 11 included studies, seven RCTs were classified as having some concerns regarding the risk of bias [59–66]. One study was classified as high risk [64], and the remaining three RCTs were evaluated as having a low risk of bias [67–69].

**Table 1:** Characteristics and demographic data of the included studies

Authors, Year	No. of patients	No. of specimens/ sinus	Male/ females	Smoker	Mean age, Years ± SD (Range)	Follow up period (months)	HA/TCP ratio	Comparative materials	Residual bone height (mm), Mean ± SD (Range)			
									BCP	Comparative materials		
Cordaro et al., 2008 [59]	36	32	NR	≤ 10/ day	(18–70)	5.3 to 8	60:40	Xeno (DBB)	5.1 ± 1.1			4.9 ± 0.8
Danesh-Sani et al., 2016 [67]	10	20	NR	Non-smoker	7.3 (25–72)	6 to 8	60:40	AB	< 5			
Froum et al., 2008 [60]	12	21	NR	≤ 10/ day	NR	6 to 8	60:40	Xeno (DBB)	< 5			
Jelusic et al., 2017 [61]	60	60	32/ 28	≤ 10 per day	(18–80)	6	60:40	TCP	2.73 ± 1.06		2.78 ± 1.31	
Kolerman et al., 2017 [68]	13	26	6/ 7	≤ 10 per day	58 (43–68)	9	60:40	AL (FDBA)	< 5			
Kolerman et al., 2019 [62]	13	26	7/ 6	≤ 10 per day	57.8 ± 6.4 (43–68)	9	60:40	AL (FDBA)	(1–5)			
Lindgren et al., 2009 [63]	11	22	5/ 6	≤ 10 per day	67 (50–79)	8	60:40	Xeno (DBB)	< 5			
Oh JS et al., 2019 [64]	56	60	33/ 25	Non-smoker	54.3 (20–69)	6	60:40	Xeno (DBB)	3.8 (2.2–5.8)			
Schmitt et al., 2013 [65]	30	53	13/ 17	< 5 per day	(38–79)	5	60:40	AB, Xeno (DBB), AL (FDBA)	2.29 ± 1.07	2.17 ± 1.27 (AB), 2.47 ± 0.99 (Xeno), 2.58 ± 0.9 (AL)		
Tosta et al., 2013 [69]	30	30	NR	Non-smoker	(18–70)	9	60:40	AB	4.1 (3–6)			
Arunjaroensuk et al., 2024 [66]	12	12	NR	NR	55.77 ± 11.48 (26–76)	6–10	70:30	Xeno (DBB)	3 or less			

SD: standard deviation; NR: not reported; BCP: biphasic calcium phosphate; Xeno: Xenografts; DBB: deproteinized bovine bone; AB: Autografts; TCP: beta tricalcium phosphate; AL: Allografts; FDBA: freeze-dried bone allograft

**Table 2:** Treatment outcomes of the included studies

Authors, Year	No. of specimens/ sinus	Comparative materials	Percentages of new bone formation, Mean ± SD		Percentages of residual material, Mean ± SD		Survival rates of implant, Mean ± SD (Range)	
			BCP	Comparative materials	BCP	Comparative materials	BCP	Comparative materials
Cordaro et al., 2008 [59]	32	Xeno (DBB)	21.6 ± 10	19.8 ± 7.9	26.6 ± 5.2	37.7 ± 8.5	NR	NR
Danesh-Sani et al., 2016 [67]	20	AB	28.2 ± 8.4	36.8 ± 11.5	32.9 ± 8.1	4.8 ± 2.4	100	100
Froum et al., 2008 [60]	21	Xeno (DBB)	28.4 ± 23.8	22.3 ± 6.4	28.4 ± 14.9	26 ± 9.7	NR	NR
Jelusic et al., 2017 [61]	60	TCP	38.42 ± 12.61	36.16 ± 19.37	32.66 ± 12.57	30.26 ± 11.7	100	98.9
Kolerman et al., 2017 [68]	26	AL (FDBA)	24 ± 6.8	27.5 ± 8.1	25.4 ± 5.5	12.5 ± 8.1	NR	NR
Kolerman et al., 2019 [62]	26	AL (FDBA)	30 ± 11 (ROI 1) 23.5 ± 9.9 (ROI 2) 22.2 ± 13.6 (ROI 3) 14.3 ± 0.6 (ROI 4)	31 ± 9.5 (ROI 1) 27.7 ± 11.2 (ROI 2) 25.1 ± 9.1 (ROI 3) 21.1 ± 12.8 (ROI 4)	21.9 ± 9.9 (ROI 1) 27.7 ± 6.6 (ROI 2) 27.6 ± 11.3 (ROI 3) 34.4 ± 1.3 (ROI 4)	7.1 ± 6.6 (ROI 1) 9.1 ± 10.3 (ROI 2) 12.4 ± 10.6 (ROI 3) 28.3 ± 8.2(ROI 4)	NR	NR
Lindgren et al., 2009 [63]	22	Xeno (DBB)	41.1 ± 9.8	41.6 ± 14	NR	NR	NR	NR
Oh JS et al., 2019 [64]	60	Xeno (DBB)	28.84 ± 7.94	25.13 ± 9.56	26.99 ± 9.16	32.19 ± 11.73	100	100
Schmitt et al., 2013 [65]	53	AB, Xeno (DBB), AL (FDBA)	30.28 ± 2.16	42.74 ± 2.1 (AB), 24.9 ± 5.67 (Xeno), 35.41 ± 2.78 (AL)	15.82 ± 2.08	21.36 ± 4.83	NR	NR
Tosta et al., 2013 [69]	30	AB	33.7 ± 8.08 (ROI 1) 26.68 ± 3.92 (ROI 2)	41.03 ± 4.62 (ROI 1) 38.63 ± 7.52 (ROI 2)	NR	NR	100	100
Arunjaroensuk et al., 2024 [66]	12	Xeno (DBB)	31.43 ± 14.36	30.09 ± 9.86	40.76 ± 16.05	45.06 ± 17.19	NR	NR

SD: standard deviation; NR: not reported; BCP: biphasic calcium phosphate; Xeno: Xenografts; DBB: deproteinized bovine bone; AB: Autografts; TCP: beta tricalcium phosphate; AL: Allografts; FDBA: freeze-dried bone allograft; ROI: region of interest

### Network meta-analysis (NMA)

**Fig. 3:**
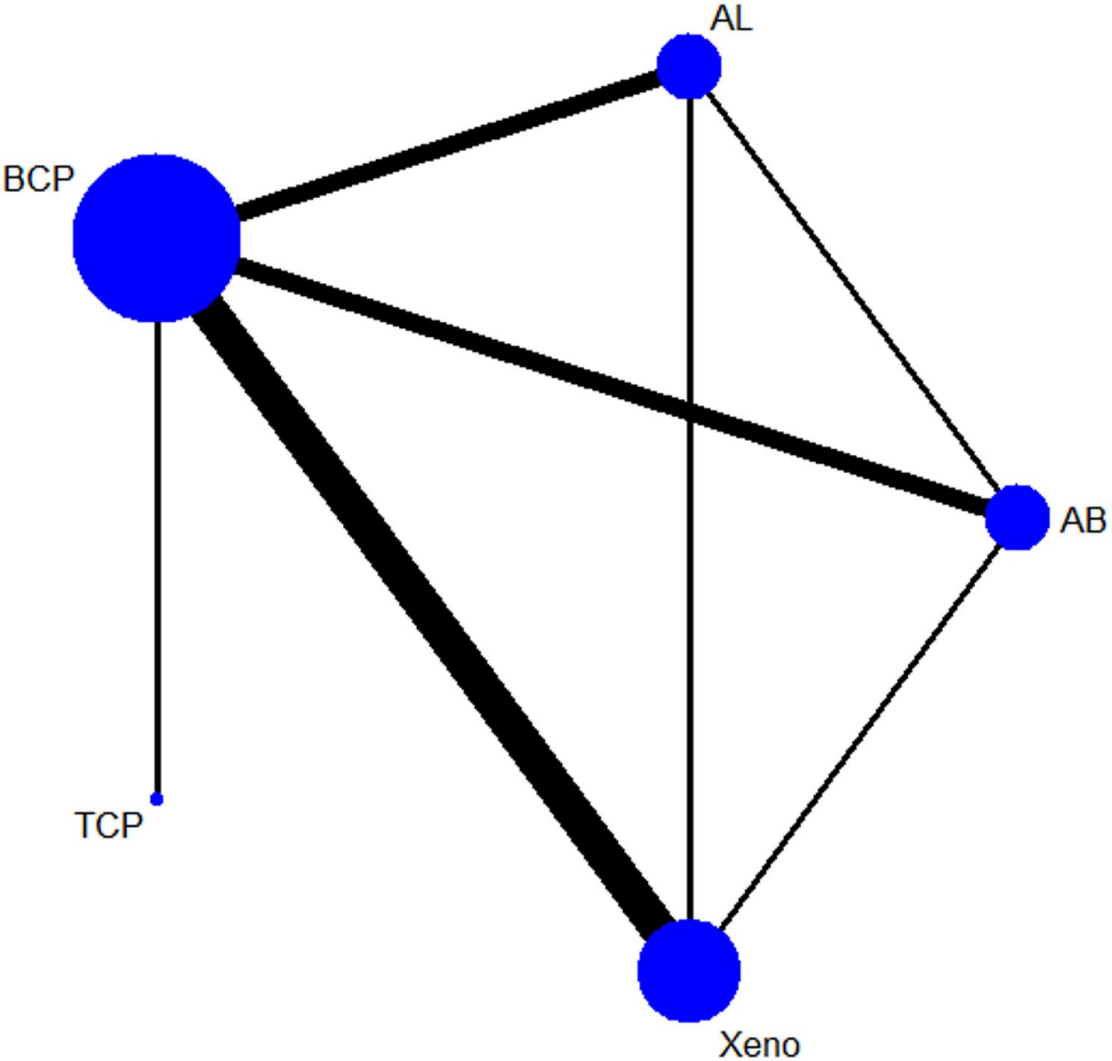
Network map of new bone formation outcomes, presenting autografts (AB), allografts (AL), beta tricalcium phosphate (TCP), BCP, and xenografts (Xeno) as the BSM used in the NMA. BCP and Xeno are the most frequently compared BSM

NMA was conducted only for new bone formation, as the results for residual material were insufficient for analysis. Figure 3 illustrates the largest circle representing BCP, indicating the primary focus on this material. Xenografts also show a significant presence, suggesting frequent comparisons with other materials. The thickness of the edges indicates a greater frequency of studies conducted between BCP and Xeno.

### New bone formation (Primary outcome)

**Fig. 4:**
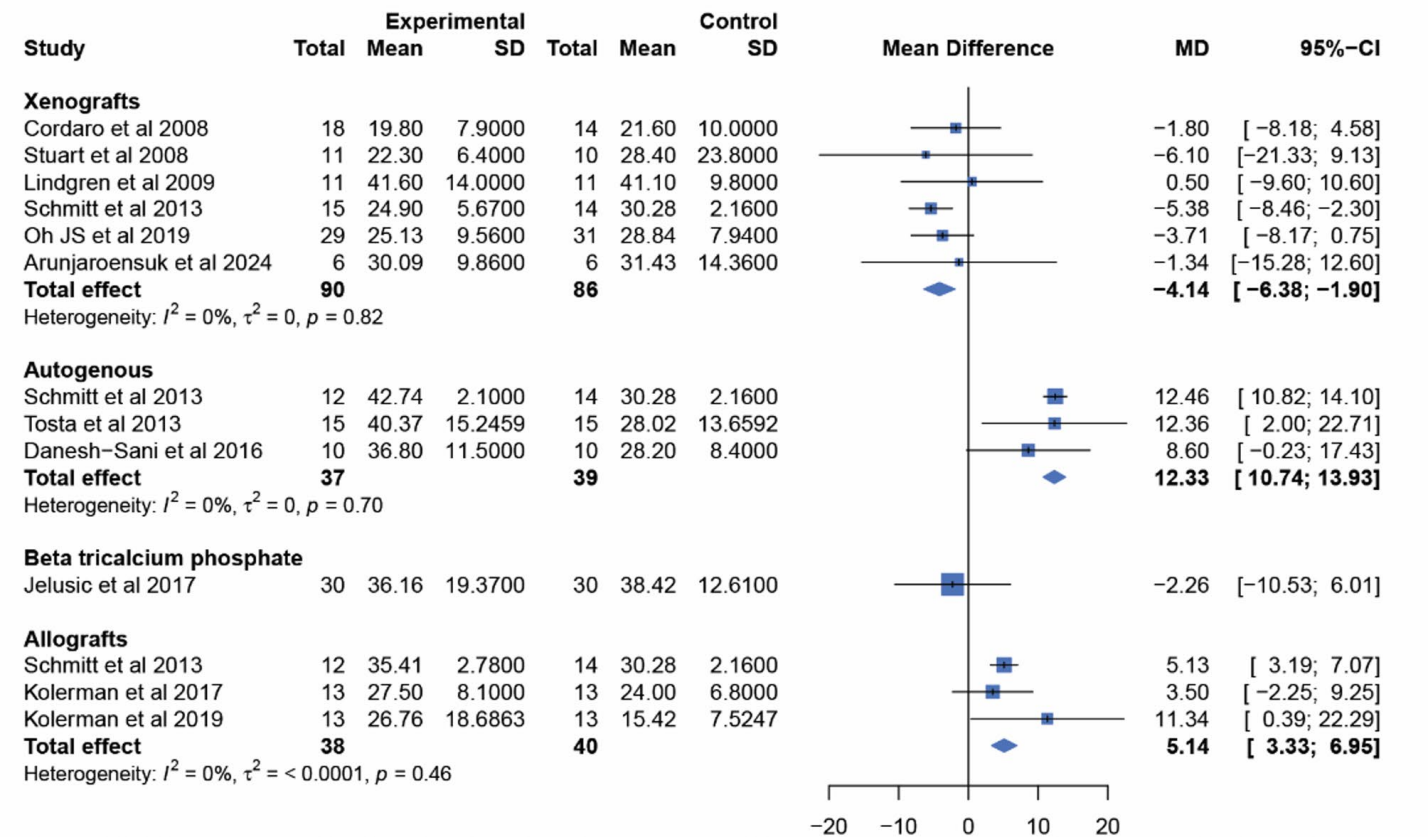
Forest plot comparing the effectiveness of each BSM in new bone formation to that of BCP

The global inconsistency test showed no evidence of inconsistency in data analysis (*p*-value = 0.6153). The NMA found that both AB and AL had a significantly greater new bone formation outcomes compared to BCP. AB showed the highest increase in new bone formation, with 12.33% more than BCP (95% CI: 10.74, 13.93). AL also outperformed BCP, showing 5.14% more new bone formation (95% CI: 3.33, 6.95), while Xeno had an inferior amount of new bone formation compared to BCP (-4.14%; 95% CI: -6.38, -1.90). There was no statistically significant difference between TCP and BCP (-2.26%; 95% CI: -10.53, 6.01) (Fig. 4).

**Fig. 5:**
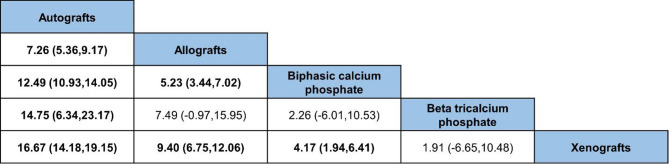
League table shows network-estimated, weighted mean difference percentage of new bone formation using different BSM

The NMA also presented the outcomes when the BSM were compared against each other. AB was found to have the greatest amount of new bone formation compared to the other BSM. AL showed superior bone volume over both BCP and Xeno, while no significant difference was observed when compared to TCP. A statistically significant difference favoring BCP was only found in comparison to Xeno (Fig. 5).

**Fig. 6:**
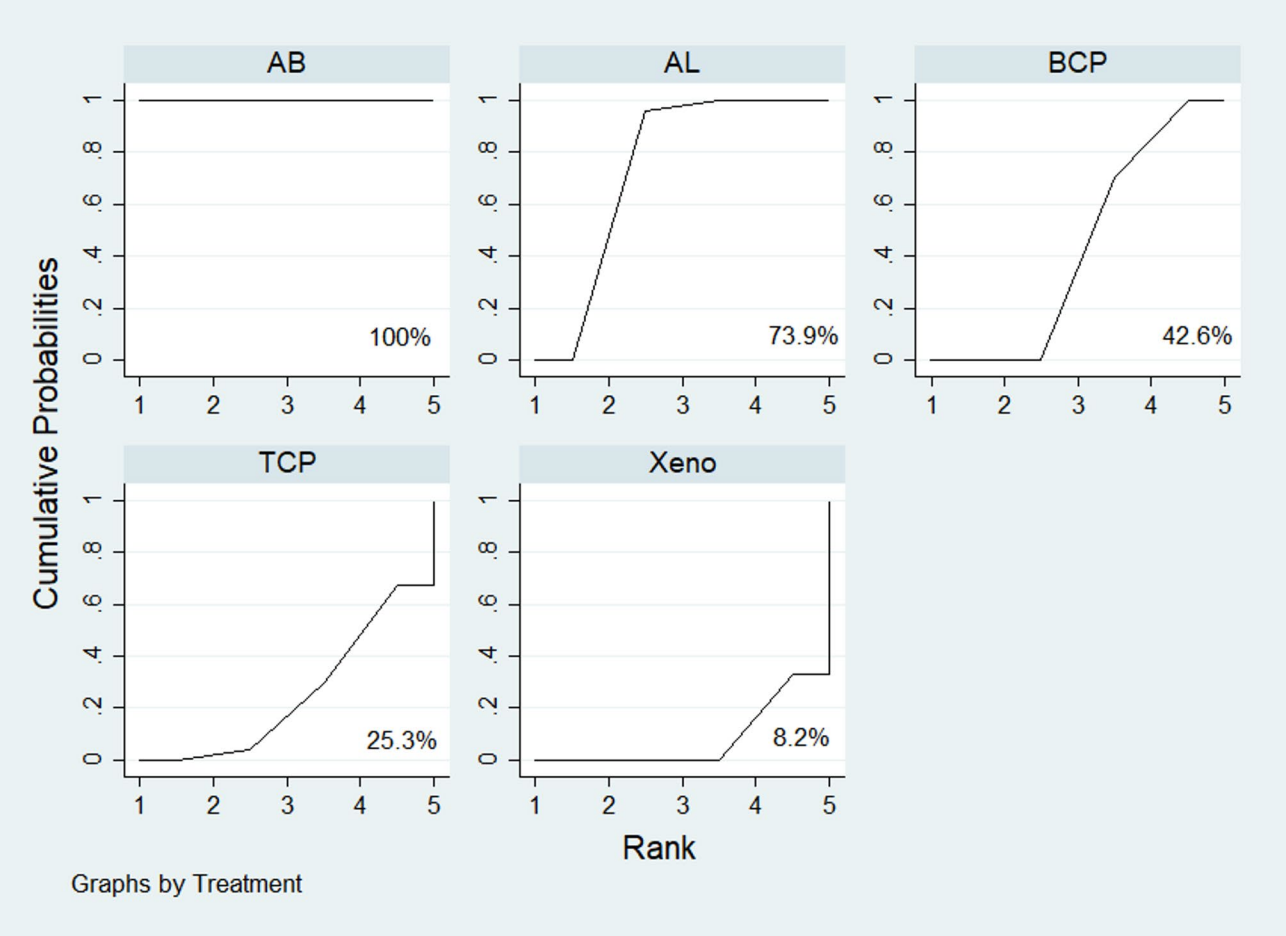
The SUCRA graph represents the probability that each BSM is among the best materials for new bone formation

The probability hierarchy of interventions was ranked using the surface under the cumulative ranking curve (SUCRA). Figure 6 shows that AB had the highest SUCRA value, indicating the greatest likelihood of being ranked first compared to the other BSM. AL, BCP, TCP, and Xeno were ranked in descending order after AB.

**Fig. 7:**
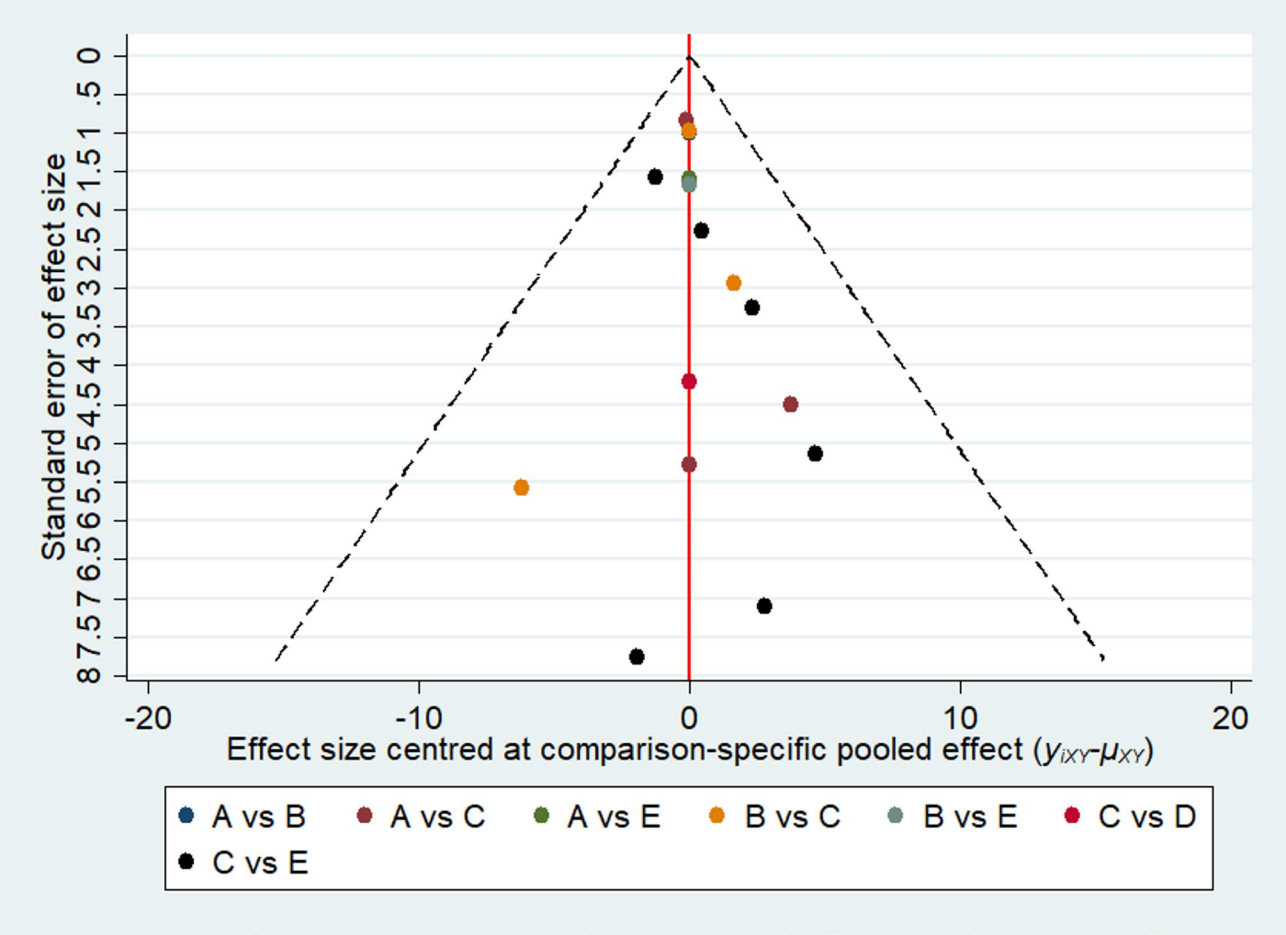
The comparison-adjusted funnel plot illustrates small-study effects and publication bias for new bone formation across all comparisons. Different comparisons are represented by different colors (A: AB, B: AL, C: BCP, D: TCP, E: Xeno)

The comparison-adjusted funnel plot was evaluated and showed no evidence of publication bias and small-study effects (Fig. 7).

### Residual graft material (Secondary outcome)

**Fig. 8:**
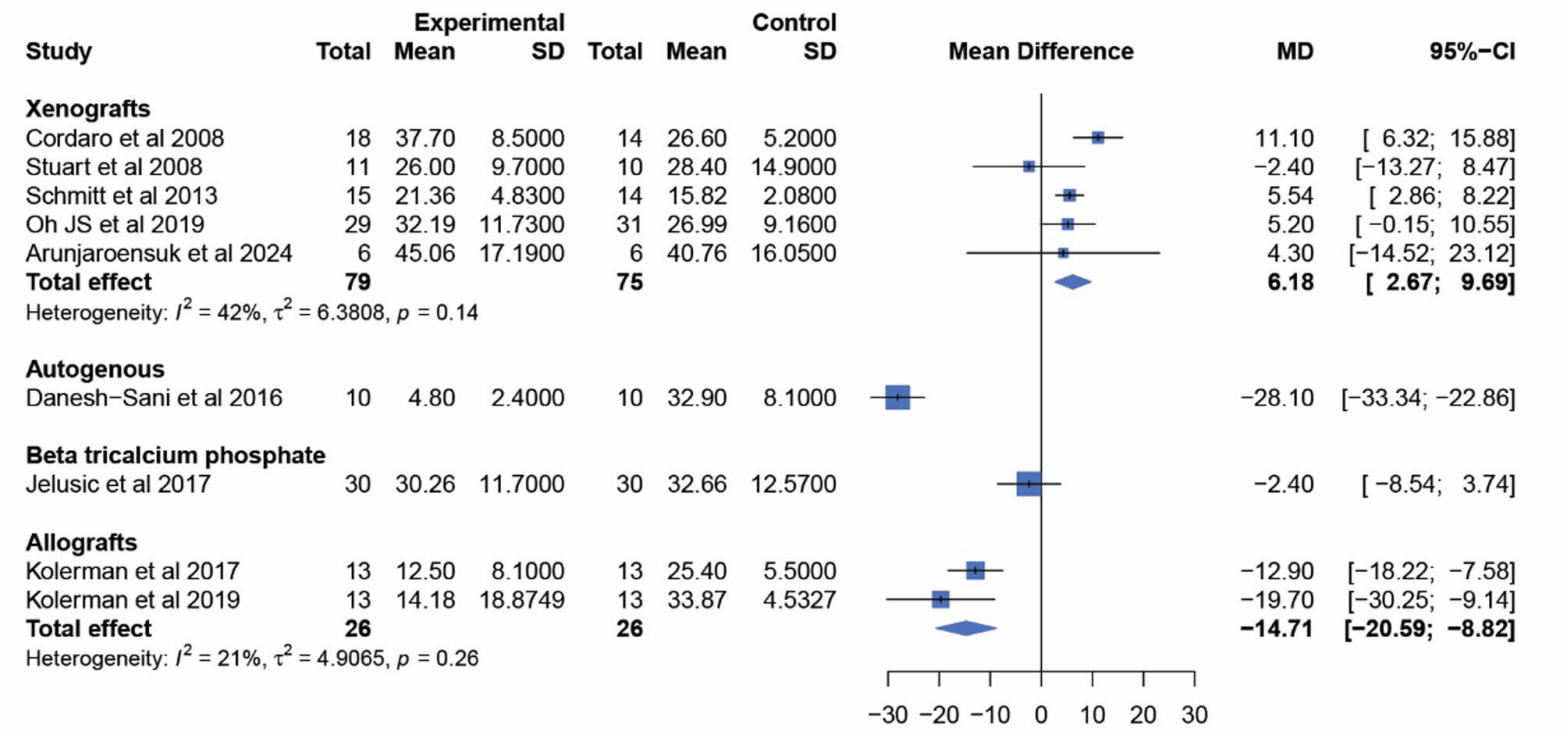
Forest plot comparing the residual graft materials of each BSM to BCP

Due to the inability to conduct NMA, pairwise meta-analyses using random effects for all directly compared interventions are presented, as shown in Fig. 8. When compared to BCP, Xeno had the highest amount of residual graft materials (6.21%; 95% CI: 2.81, 9.61). This was followed by AL and AB, which had residual graft materials of -14.71% (95% CI: -20.59, -8.82) and − 28.1% (95% CI: -33.34, -22.86), respectively. TCP showed no statistically significant difference from BCP in terms of residual graft materials (-2.4%; 95% CI: -8.54, 3.74). The pairwise meta-analysis included only nine studies. Lindgren et al. [63] and Tosta et al. [69] were excluded due to, respectively, the lack of reported standard deviations (which prevented conducting a meta-analysis) and the absence of reports on residual graft materials. Only the results for Xeno and BCP were reported from the four BSM in the study by Schmitt et al. [65], which resulted in the inability to analyze the results for AB and AL from this publication.

## Discussion

Systematic reviews summarize the outcome of various studies selected regarding defined inclusion criteria and focusing on a certain topic to answer a pre-defined focused question. Meta-analyses statistically combine data obtained from these independent studies included into systematic reviews to support decision making regarding the focused question. Network meta-analyses extend this approach by offering the opportunity to compare different therapeutic options using data from studies that actually have not been directly compared earlier, while also providing a ranking list of interventions. In this study, direct comparisons between BCP and other BSM were limited, making this approach particularly valuable, as the NMA enabled broader integration of available evidence and facilitated indirect comparisons that would not have been feasible with traditional pairwise meta-analysis.

This network meta-analysis reveals that AB used for sinus floor elevation and augmentation procedures is the graft material performing superior compared to all other BSM. Nevertheless, AL, Xeno, Alloplastic materials and BCP as well used due to their osteoconductive properties for sinus floor augmentations, revealed sufficient bone formation following different healing period durations ranging between five months (e.g., Schmitt et al. [65] and ten months (e.g., Arunjaroensuk et al. [66]). Use of BSM instead of autogenous bone grafts can also prevent donor site morbidity.

This NMA included data from 11 RCTs on new bone formation and 9 RCTs regarding residual graft materials, covering a healing period of five to ten months. The BSM used in this study were AB, AL, BCP, TCP, and Xeno, with a total of 362 sinus specimens from 283 patients. For the new bone formation outcomes, no evidence of inconsistency was found, allowing the analysis to proceed with the consistency model. Additionally, heterogeneity in all pairwise comparisons, except for TCP, showed I² values of 0%. across all comparisons indicating minimal variability between studies. In this review, I² values below 25% were considered indicative of low heterogeneity [36], reflecting minimal variability between studies. Both the pairwise meta-analysis and NMA consistently indicated that, when compared to BCP, AB and AL showed significantly greater effects on new bone formation, while Xeno demonstrated inferior effects compared to BCP. The effects of TCP, although appearing to be inferior to BCP, were inconclusive due to the presence of only a single study. Moreover, the NMA allowed us to evaluate the outcomes when the BSM were compared against each other. According to the results, AB had the greatest effect and the highest likelihood of being ranked first for new bone formation, while our focus BSM, BCP, ranked third.

The new bone formation effects of AB, AL, and Xeno, when compared to BCP, appear to align with their characteristics. AB and AL, with both osteoinductive and osteoconductive properties [6, 7, 13], enhance the bone-forming capacity of grafts. Additionally, AB possesses osteogenic properties [6, 7], which likely contributes to its superior ranking when compared to BCP and other biomaterials. Although BCP also exhibits osteoinductive and osteoconductive effects [13, 22], its osteoinductive potential is diminished by the inclusion of HA, which lowers the Ca^2+^ concentration released from TCP content [13, 22]. The presence of osteoinductive properties appears to play a critical role in bone formation, as evidenced by Xeno, which possesses only osteoconductive properties [7, 13], and ranks the lowest in promoting bone generation. Despite lacking HA content, TCP does not demonstrate significantly higher bone formation compared to BCP. However, due to the limited data from a single study, definitive conclusions regarding its superiority or inferiority cannot be drawn, highlighting the need for further research with larger sample sizes.

Residual graft materials were considered secondary outcomes and were additionally retrieved during the data extraction process. Since all included publications reported the percentage of new bone formation, the absence of data on residual graft materials did not serve as an exclusion criterion. As a result, these studies were included, but this resulted in insufficient data to conduct NMA for the outcomes of residual graft materials. Aside from the lack of reported standard deviations in one study [63], the main problem and limitation in the data analysis was the inability of the studies themselves to report the percentage of residual graft material or to report on all the BSMs used. AB was particularly affected, as the two studies that used AB as a comparison [65, 69] reported an inability to detect residual AB after bone healing. Consequently, only one study was available for analysis in the pairwise meta-analysis. Given these limitations, only AL and Xeno provided statistical results regarding residual graft materials. HA is the primary component of Xeno, whereas BCP contains only 60–70% HA. This composition makes Xeno solely osteoconductive and limits its resorption rate due to the inherent properties of HA [7, 13, 14]. In contrast, the lower proportion of HA in BCP makes it more biodegradable [13, 16], allowing bone cells to more easily replace it with new bone. Human-derived BSM, AL, have a structure and composition similar to natural bone, resulting in a resorption rate more comparable to that of human bone than BCP.

However, a key limitation of this study is the specific BCP composition used in the analysis, predominantly with a 60:40 HA to TCP ratio. Since the osteoconductive activity and biodegradability of BCP can be influenced by adjusting the HA/ TCP ratio [13, 19], different proportions of BCP could yield results that differ from those observed in this study, and their effects should be further investigated. Although one study used a 70:30 HA to TCP ratio [66], the limited data precluded analysis of ratio differences and subgroup comparisons. Consequently, the present review combined data from all eligible studies to evaluate the general effectiveness of BCP, while acknowledging the compositional heterogeneity.

To address this limitation, we conducted a sensitivity analysis restricted to studies using only the 60:40 HA/TCP formulation. The results, presented in Supplementary Figure S1, revealed I² values of 0% across all comparisons, indicating minimal heterogeneity and consistency with the primary findings. This suggests that the inclusion of a single 70:30 BCP study did not substantially influence the overall results. Nonetheless, these findings should be interpreted primarily in the context of 60:40 BCP, and caution is warranted when generalizing to BCPs with alternative HA/TCP ratios, which may differ in clinical performance.

The residual bone height of the maxillary sinus floor may present a limitation in this study, as papers examining cases with residual bone heights ranging from 1 to 6 mm were included. Previous studies by Reich et al. [70] and Taschieri et al. [71] have indicated that increased recipient bone height is associated with enhanced bone regeneration capacity, likely due to a greater availability of mesenchymal stem cells that contribute to bone regeneration. Conversely, several studies report no correlation between residual bone height and new bone formation [72–75], instead highlighting sinus width as a more significant factor. With osteogenic and vascular components primarily originating from the sinus walls and floor, a large sinus size may limit osteogenic potential due to the increased distance to the graft site [72, 74, 76–78]. Given this variability, the generalizability of the findings may be limited to cases with residual bone height ≤ 6 mm. Furthermore, the limited reporting of recipient site width in the included studies constrained the ability to analyze its effect on new bone formation in this analysis. Additionally, splitting the healing period into subgroups could hinder data analysis; therefore, further fragmentation of the data based on healing period was not performed. Consequently, the influence of sinus morphology and healing period on the histomorphometric results could not be analyzed in this study. Further research is needed to determine how variations in residual bone height below 6 mm affect bone regeneration.

Although several studies and systematic reviews have reported implant survival rates exceeding 90% for various BSMs, data specifically addressing implant survival in sites augmented with BCP remain limited [23 – [25, 61, 64, 67, 69]. Most studies included in this review did not report implant survival outcomes, making it impossible to draw meaningful conclusions or perform a quantitative analysis. This lack of data should be recognized as a significant limitation.

Smoking status was initially intended to be included as a subgroup in the sensitivity analysis, given its well-documented impact on bone healing. Smoking adversely affects new bone formation and contributes to increased bone resorption and impaired regenerative capacity [79–81]. However, this analysis could not be conducted due to the inconsistent reporting across the included studies. Nevertheless, most studies included only non-smokers or individuals who smoked fewer than 10 cigarettes per day, acknowledging the known effects of smoking on bone regeneration and implant survival rates [82–86]. As a result, the results of this review should be interpreted with caution when applied to heavy smokers or patients who smoke more than 10 cigarettes per day.

Importantly, the lack of long-term follow-up data (5 to 10 years) for BCP limits the ability to assess the durability of bone formation after augmentation. To the best of our knowledge, the longest follow-up period evaluating BCP in comparison to DBB is six years, which demonstrated over 90% implant survival rates and less than 1 mm of marginal bone loss in the BCP group [24, 25, 87, 88]. This absence of broader long-term data restricts conclusions regarding the sustained clinical effectiveness of BCP. Further research with extended follow-up periods is warranted to address this gap.

Among other studies with relatively longer follow-ups, a two-year study reported no significant differences in implant stability or survival rates between HA and BCP (20:80), though BCP exhibited less volumetric reduction after augmentation [89]. One- and three-year follow-up studies by Lindgren et al. [57, 90] also showed no difference in implant survival rates between BCP (60:40) and DBB, with survival rates over 95%. Similarly, histomorphometric outcomes of BCP and DBB at the three-year follow-up showed no significant differences [90]. However, the number of long-term follow-up studies investigating BCP alone or in comparison with other BSMs remains scarce, highlighting the need for further research in this area.

Moreover, a manual search of specific journals was not performed, grey literature was excluded from this study, and only studies published in English were included. These factors may have limited the identification of additional eligible studies and introduced potential for publication bias and language bias. However, visual inspection of funnel plots did not reveal clear evidence of publication bias, suggesting that the exclusion of grey literature may not have significantly affected the overall results. Nonetheless, the possibility of publication bias cannot be completely excluded due to the inherent limitations of funnel plot analyses, especially with a limited number of studies.

While this review provides histomorphometric outcomes and demonstrates that BCP yields statistically comparable results to other materials, the clinical significance of these findings should be interpreted with caution. Currently, there are no established thresholds to define what magnitude of difference in new bone formation constitutes a clinically meaningful outcome. Moreover, economic considerations including cost-effectiveness remain underexplored. Future research is needed to evaluate the cost-effectiveness of bone substitute materials in parallel with their clinical performance. Given these limitations, the choice of grafting materials should not rely solely on superior histomorphometric outcomes but should also take into account broader factors, including patient-specific considerations (such as economic, ethical, or religious factors) and site-specific conditions, to provide more comprehensive treatment planning.

Despite these limitations, new bone formation outcomes showed no heterogeneity according to the results of the global inconsistency test. Thus, despite variations in the healing period and residual bone height, the effect sizes and outcomes can be interpreted consistently. Furthermore, these results should generally be applicable.

## Conclusion

Within the limitations of this analysis, it can be concluded that AB used for sinus floor elevation and augmentation procedures is the graft material performing superior compared to all other BSM regarding percentage of bone formation. Nevertheless, AL, Xeno and BCP as well used due to their osteoconductive properties for sinus floor augmentations revealed sufficient bone formation following different healing period durations. While human-derived BSM continues to show superior bone regeneration, BCP outperforms Xeno in terms of both bone formation and residual graft material. Based on these findings, BCP may be considered a practical alternative in clinical scenarios where the use of AB or AL is limited. However, given the absence of defined thresholds for clinically meaningful bone formation and the lack of cost-effectiveness and long-term implant survival data, treatment decisions should be made not only by histomorphometric outcomes but also by broader clinical, economic, and patient-specific factors.

## Electronic supplementary material

Below is the link to the electronic supplementary material.

Supplementary Material 1

## Data Availability

No datasets were generated or analysed during the current study.

## Abbreviations

AB: Autografts
AL: Allografts
BCP: Biphasic calcium phosphate
BMP: Bone morphogenic proteins
BSM: Bone substitute materials
DBB: Deproteinized bovine bone
DFDBA: Demineralized freeze-dried bone allografts
FDBA: Freeze-dried bone allografts
FFB: Fresh-frozen bone
HA: Hydroxyapatite
NMA: Network meta-analysis
NR: Not reported
RCTs: Randomized controlled trials
ROI: Region of interest
SD: Standard deviation
TCP: Beta tricalcium phosphate
Xeno: Xenografts
