# Supplementary material for: New bone formation of biphasic calcium phosphate bone substitute material: a systematic review and network meta-analysis of randomized controlled trials (RCTs)
Source: Int J Implant Dent. 2025 Jul 15;11:47. doi: 10.1186/s40729-025-00636-4 (PMC12263524; doi:10.1186/s40729-025-00636-4)
Supplement: Supplementary file 1 — Supplementary Material 1 [file 40729_2025_636_MOESM1_ESM.docx]

**Supplementary materials**

Table S1. Search algorithms

Table S2. Reasons for excluded studies

Figure S1. Sensitivity analysis of newly formed bone, limited to studies using biphasic calcium phosphate (BCP) with a 60:40 HA/TCP ratio.

Figure S2. Sensitivity analysis of residual graft material, limited to studies using biphasic calcium phosphate (BCP) with a 60:40 HA/TCP ratio.

**Table S1. Search algorithms**

| Database | Search string |
| --- | --- |
| MEDLINE (via PubMed) | (((((((((((((((Alveolar Ridge Augmentations) OR (Ridge Augmentation)) OR (Augmentation)) OR (Sinus Floor Augmentations)) OR (Maxillary Sinus Augmentation)) OR (sinus lift)) OR (sinus floor elevation)) OR (sinus elevation)) OR (lateral sinus lift)) OR (sinus lateral window)) OR (lateral window technique)) OR (GBR)) OR (guided bone regeneration)) OR (bone augmentation)) AND ((((biphasic calcium phosphate) OR (hydroxyapatite-beta tricalcium phosphate)) OR (BCP)) OR (HA/β-TCP))) AND (((histomorphometric) OR (bone histomorphometry)) OR (histomorphometric analysis)) |
| Cochrane | ("alveolar ridge augmentation" OR "ridge augmentation" OR "augmentation" OR "sinus floor augmentation" OR "maxillary sinus augmentation" OR "sinus lift" OR "sinus floor elevation" OR "sinus elevation" OR "lateral sinus lift" OR "sinus lateral window" OR "lateral window technique" OR "GBR" OR "guided bone regeneration" OR "bone augmentation") AND  ("biphasic calcium phosphate" OR "hydroxyapatite-beta tricalcium phosphate" OR "BCP" OR "HA/β-TCP") AND  ("histomorphometric" OR "bone histomorphometry" OR "histomorphometric analysis") |
| Scopus | ("alveolar ridge augmentation" OR "ridge augmentation" OR "augmentation" OR "sinus floor augmentation" OR "maxillary sinus augmentation" OR "sinus lift" OR "sinus floor elevation" OR "sinus elevation" OR "lateral sinus lift" OR "sinus lateral window" OR "lateral window technique" OR "GBR" OR "guided bone regeneration" OR "bone augmentation") AND  ("biphasic calcium phosphate" OR "hydroxyapatite-beta tricalcium phosphate" OR "BCP" OR "HA/β-TCP") AND  ("histomorphometric" OR "bone histomorphometry" OR "histomorphometric analysis") |
| Embase | ("alveolar ridge augmentation" OR "ridge augmentation" OR "augmentation" OR "sinus floor augmentation" OR "maxillary sinus augmentation" OR "sinus lift" OR "sinus floor elevation" OR "sinus elevation" OR "lateral sinus lift" OR "sinus lateral window" OR "lateral window technique" OR "GBR" OR "guided bone regeneration" OR "bone augmentation") AND  ("biphasic calcium phosphate" OR "hydroxyapatite-beta tricalcium phosphate" OR "BCP" OR "HA/β-TCP") AND  ("histomorphometric" OR "bone histomorphometry" OR "histomorphometric analysis") |

**Table S2. Reasons for excluded studies**

| Study (First Author, Year) | \| Reason for Exclusion \| \| --- \| |
| --- | --- | --- |
| Flichy-Fernández et al., 2019 | Used combined materials |
| Prins et al., 2016 | Used combined materials |
| Taschieri et al., 2016 | Used combined materials |
| Wagner et al., 2012 | Used combined materials |
| Shayesteh et al., 2008 | Used combined materials; No comparative material |
| Kim et al., 2015 | Used combined materials; Insufficient data for NMA |
| Artzi et al., 2008 | No comparative material |
| Mangano et al., 2013 | No comparative material |
| Ohayon, 2014 | No comparative material |
| Frenken et al., 2010 | No comparative material |
| Mangano et al., 2015 | No comparative material |
| Helder et al., 2018 | Both groups used BCP |
| Sokolowski et al., 2020 | Insufficient data for NMA |
| Kraus et al., 2020 | Insufficient data to conduct NMA |
| La Monaca et al., 2018 | Insufficient data to conduct NMA |
| de Lange et al., 2014 | Insufficient data to conduct NMA |
| Annibali et al., 2015 | Insufficient data to conduct NMA |
| Velasco-Ortega et al., 2021 | Used materials other than BCP |
| Tomas et al., 2023 | No lateral sinus augmentation |
| Iezzi et al., 2012 | Not an RCT |
| Lindgren et al., 2012 | Not an RCT |

Note: Some studies were excluded for more than one reason. NMA: network meta-analysis; BCP: biphasic calcium phosphate; RCT: randomized control trial

**Figure S1. Sensitivity analysis of newly formed bone, limited to studies using biphasic calcium phosphate (BCP) with a 60:40 HA/TCP ratio.
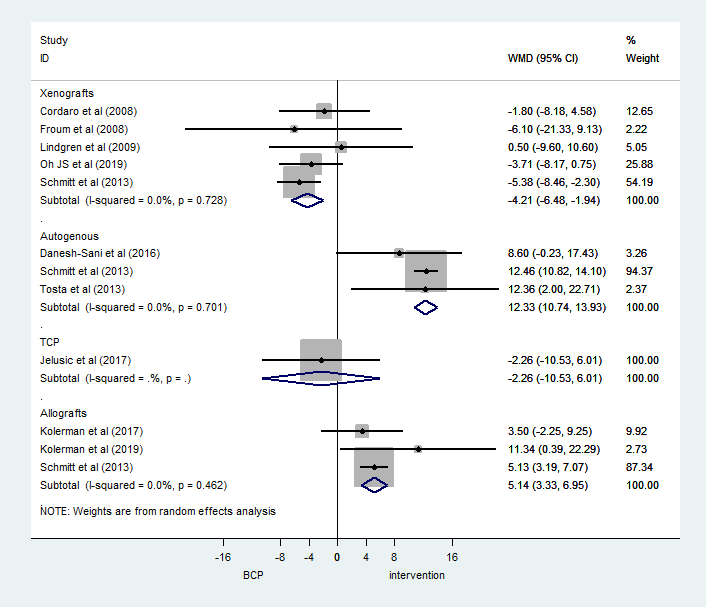
**

**Figure S2. Sensitivity analysis of residual graft material, limited to studies using biphasic calcium phosphate (BCP) with a 60:40 HA/TCP ratio.**

**
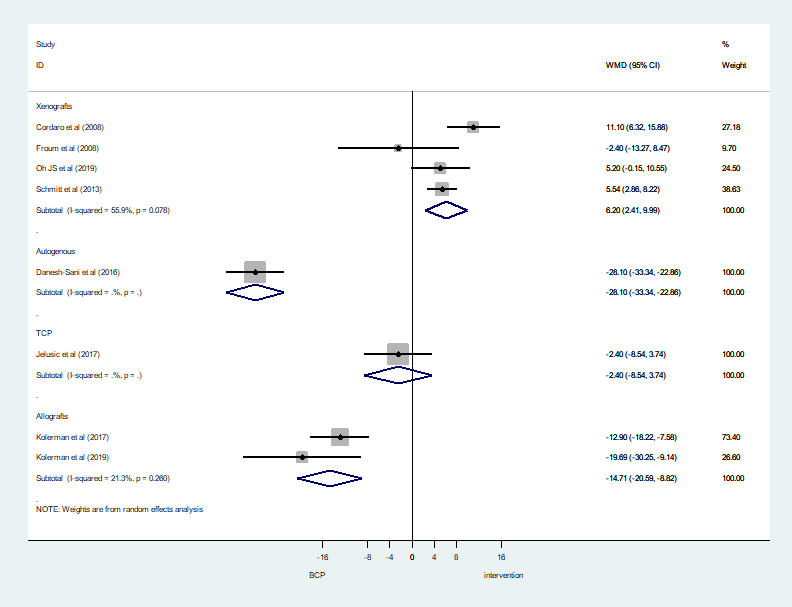
**
